# Long-term outcomes of pT1 rectal cancer after transanal endoscopic surgery: again, a word of caution on high local recurrence — a cohort study

**DOI:** 10.1007/s00384-025-05038-x

**Published:** 2026-01-03

**Authors:** Xavier Serra-Aracil, Cristina Gener-Jorge, Anna Nonell, Joan Carles Ferreres-Piñas, Beatriz Espina, Alex Casalots, Aleidis Caro-Tarragó

**Affiliations:** 1 Department of Surgery, Universitat Autònoma de Barcelona, Barcelona, Spain; 2 Department of Morphological Sciences, Universitat Autònoma de Barcelona, Barcelona, Spain; 3 Parc Taulí Hospital Universitari, Department of Surgery. Institut d’Investigació i Innovació Parc Taulí (I3PT-CERCA), Universitat Autònoma de Barcelona, Sabadell, Spain; 4 Parc Taulí Hospital Universitari, Department of Pathology. Institut d’Investigació i Innovació Parc Taulí (I3PT-CERCA), Universitat Autònoma de Barcelona, Sabadell, Spain; 5 Hospital de La Santa Creu I Sant Pau, Autonomous University of Barcelona (UAB). Institut de Recerca Sant Pau (IR Sant Pau), Barcelona, Spain; 6 CLILAB Diagnostics, Department of Pathology, Consorci del Laboratori Intercomarcal de L’Alt Penedés I El Garraf, Barcelona, Spain; 7 Department of Surgery, Hospital Universitari Joan XXIII, Tarragona, Spain

**Keywords:** Transanal endoscopic microsurgery (TEM), Early rectal cancer, Local excision, Local recurrence, Prognosis of T1 rectal adenocarcinoma, Oncological outcome, TME

## Abstract

**Purpose:**

Recent evidence suggests that the local recurrence (LR) rate after local excision of pT1 rectal adenocarcinoma may be higher than previously estimated, particularly in large cohorts with extended follow-up. This study aimed to evaluate the LR rate and long-term oncological outcomes in patients with pT1 rectal adenocarcinoma treated with transanal endoscopic surgery (TES).

**Method:**

Observational cohort study including 824 consecutive patients who underwent TES at a single tertiary center between 2004 and 2021. Among them, 104 patients (12.6%) were diagnosed with pT1 rectal adenocarcinoma. Patients were excluded if they had received neoadjuvant or adjuvant chemoradiotherapy, had non-rectal tumors, a follow-up of less than 40 months, or were treated with surgical techniques other than TES.

**Results:**

With a median follow-up of 91 months (IQR: 84), 17 patients (16.3%) developed LR and 14 (13.5%) developed distant recurrence. Five-year rectal cancer–specific and overall survival rates were 95% and 74%, respectively. Among the 88 patients without histopathological or surgical high-risk factors, 13 (14.8%) experienced LR and 9 (10.2%) distant recurrence. Their five-year disease-free and overall survival rates were 95% and 74%, Multivariate analysis identified flat-ulcerated morphology as the only independent predictor of LR (OR 6.8; 95% CI 1.5–30.4; p = 0.01).

**Conclusion:**

TES for pT1 rectal adenocarcinoma resulted in a 16.3% overall LR rate, and 14.8% among patients without known risk factors, emphasizing the need for improved patient selection and novel prognostic and therapeutic tools. These findings warrant confirmation in multicenter studies with standardized criteria and prolonged follow-up.

**Supplementary Information:**

The online version contains supplementary material available at 10.1007/s00384-025-05038-x.

## Introduction

Early diagnosis of colorectal cancer has increased in recent decades due to the implementation of screening programs, allowing for the detection of tumors at early stages, such as pT1 rectal adenocarcinoma (ADK) [1, 2]. In the absence of adverse pathological features, international guidelines recommend local excision (LE) as the standard treatment, using either endoscopic or surgical techniques, such as transanal endoscopic surgery (TEM/TEO), which offer favorable functional outcomes and low morbidity rates [3].

However, in general terms, the standard treatment for rectal cancer remains TME, with or without neoadjuvant therapy, depending on the tumor stage. This radical approach is associated with considerable morbidity, long-term functional disorders, and, consequently, a significant reduction in quality of life [4, 5]

The indication for LE in early-stage colorectal cancer (CRC) represents a significant clinical challenge, as it requires accurate estimation of the risk of lymph node metastasis and LR. However, the functional benefits of LE must be carefully weighed against this potential oncological risk. In general, the incidence of lymph node metastases in pT1 CRC tumors has been reported to range from 6 to 17% [6, 7]. The clinical manifestation of this risk in the form of LR remains one of the main concerns, particularly due to the high variability in recurrence rates reported for pT1 CRC. This variability is especially evident in studies of endoscopic resection that combine colon and rectal tumors, where LR rates below 5% have been reported [8, 9]. Nevertheless, these results are not directly applicable to tumors located exclusively in the rectum, which present distinct clinical and anatomical features and, consequently, considerably higher LR rates [10–12].

Most meta-analyses evaluating LR (LR) in pT1 rectal tumors include heterogeneous series, often with small sample sizes, limited follow-up, and poorly standardized diagnostic criteria. A recent meta-analysis that included only studies with a minimum follow-up of 36 months reported an overall LR rate of 6.7% after LE of low-risk pT1 rectal cancer, and 13.6% in cases with high-risk features [10]. A more recent meta-analysis reported an LR rate of 8.2% for T1 tumors, calculated using a weighted average of the included studies. However, only one of these studies had more than 100 patients, which may affect the validity of the conclusions [12].

Another important factor to consider is that, in studies of conventional endoscopic resection, the tumors treated are often significantly smaller than those managed with TEM/TEO, which may introduce substantial bias when comparing recurrence rates. In this context, it is particularly concerning that one of the largest series published to date, by Leijtens et al. [13], reported a five-year LR rate of 22.7% in a cohort of 150 patients with pT1 rectal cancer treated with TEM/TEO.

The present study reports the outcomes from a single center with over 20 years of experience in TEM/TEO for the treatment of pT1 rectal ADK, applying consistent criteria for treatment indication, surgical technique, decision-making regarding completion total mesorectal excision (TME), and follow-up protocol.

The primary objective of this study was to analyze the long-term LR rate in patients with pT1 rectal ADK without adverse histopathological features, treated with TEM/TEO. Secondary objectives included the analysis of tumor characteristics, surgical variables, distant recurrence (DR) rate, overall survival, disease-free survival, rectal cancer–specific survival, and identification of factors associated with LR.

## Materials and methods

### Study design

This was an observational cohort study based on a consecutive series of patients who underwent TEM/ TEO. Data were prospectively collected and retrospectively analyzed. Data management was carried out using a protected digital database in Microsoft® Access 2003.

The study was approved by the institutional Clinical Research Ethics Committee (reference: 2023/5123) and conducted in accordance with the ethical principles of the Declaration of Helsinki. This manuscript was prepared following the STROBE guidelines for reporting observational studies[14]. All patients provided written informed consent.

### Patients and setting

All patients were operated on by colorectal surgeons from our institution’s Coloproctology Unit between June 2004 and December 2021. All candidates for TEM/TEO underwent a standardized preoperative evaluation protocol [15, 16]. Based primarily on the findings from endorectal ultrasound (ERUS-u-) [17] and pelvic magnetic resonance imaging (MRI-mr-), patients were classified into five groups according to preoperative indication: Group I: Curative intent in benign lesions staged as u-mrT0-1 and u-mrN0. Group II: Curative intent in well-differentiated ADK (low grade), staged as u-mrT0-1 and u-mrN0. Group III: *Consensual indication* for low-grade ADK staged as u-mrT2 and u-mrN0, in patients who declined radical surgery. Group IV: Palliative indication. Group V: Atypical indication [18]. All cases were evaluated by a multidisciplinary colorectal cancer board, which determined the most appropriate treatment strategy based on staging and current clinical guidelines [19, 20].

Inclusion criteria: Patients diagnosed with submucosal invasive ADK (pT1), according to the American Joint Committee on Cancer (AJCC) classification [21], who were treated with TEM or TEO and had a minimum follow-up of 42 months (3.5 years) from the time of surgery.

Exclusion criteria: Patients who received neoadjuvant treatment; postoperative radiotherapy or chemotherapy; pT1 malignant tumors other than adenocarcinoma; tumors located outside the rectum or LE techniques other than TEM/TEO.

### Patient preparation and surgical technique

Patients scheduled for TEM/TEO underwent antegrade mechanical bowel preparation, along with antibiotic and antithrombotic prophylaxis according to the institutional protocol [15, 16]. General anesthesia was used in most cases, although spinal anesthesia was selected by the anesthesiologist in some instances. LE was performed using either transanal endoscopic microsurgery (TEM) (Richard Wolf, Knittlingen, Germany) or transanal endoscopic operation (TEO) (Karl Storz GmbH, Tüttlingen, Germany).

The LE technique consisted of a full-thickness resection of the rectal wall using an ultrasonic scalpel, avoiding penetration into the perirectal fat, and following a standardized surgical technique [22]. When feasible, the rectal wall defect was closed with a tension-free suture. The urinary catheter was removed at the end of the procedure. Both oral intake and ambulation were initiated six hours after surgery. Over time, the length of hospital stay has progressively decreased, currently averaging 24 h or even same-day discharge, except in cases with postoperative complications [23].

### Pathological assessment and follow-up

The surgical specimen was placed and fixed on a cork board, with resection margins preserved using pins. A macroscopic description of the specimen was performed; all margins were inked, and the entire specimen was submitted for histological evaluation. It was then processed and stained with hematoxylin and eosin.

Following the pathological report, all cases were reviewed by the multidisciplinary colorectal cancer board. In patients with pT1 rectal tumors exhibiting adverse histological features—such as poor differentiation (G3 or G4), lymphatic, vascular, or perineural invasion, lymphocytic infiltration [24], or the presence of tumor budding—a second procedure was recommended to complete treatment with TME [25].

Patient follow-up was conducted by the Colorectal Surgery and Medical Oncology Units according to standard rectal cancer surveillance protocols [20].

### Variables

The *primary outcome* of the study was local LR following TEM/TEO, defined as either endoluminal recurrence or nodal recurrence within the pelvis [11].

*Secondary variables* included:

Demographic and preoperative variables: age, sex, ASA classification, histological type on preoperative endoscopic biopsy, tumor size, tumor distance from the anal verge, morphological type of the lesion, tumor location by rectal quadrant, and preoperative staging by endorectal ultrasound (ERUS) and magnetic resonance imaging (MRI), both T and N stages.

Surgical variables: surgical technique used (TEM or TEO), operative time, type of resection (en bloc or fragmented), closure of the rectal wall defect, and length of hospital stay.

Postoperative variables: 30-day morbidity, classified according to the Clavien–Dindo classification [26], and pathological findings (tumor type and size, resection margins, grade of differentiation, and adverse histological features such as lymphatic, vascular, or perineural invasion, lymphocytic infiltration, and tumor budding).

Follow-up variables included: need for completion TME, type of surgery performed, distant recurrence (DR), disease-free survival (DFS), rectal cancer–specific survival (CSS), and overall mortality. DFS was defined as the time from surgery to any local or distant recurrence, or death from any cause. CSS was defined as time from surgery to death attributable to rectal adenocarcinoma. OS was defined as time from surgery to death from any cause. DR was defined as any radiologically or histologically confirmed distant metastasis after the index local excision, with the event date set as the first documented DR.

### Statistical analysis

Data were analyzed using SPSS software, version 26 (SPSS Inc., Chicago, IL). The prospective data collection allowed for analysis with no missing values, except in certain variables related to oncological follow-up. Quantitative variables were described using means and standard deviations, or medians and interquartile ranges when normality was not met, as assessed by the Kolmogorov–Smirnov test. Categorical variables were presented as absolute frequencies and percentages.

For univariate analysis, quantitative variables between independent groups were compared using the Student’s t-test when assumptions of normality and homogeneity of variances were met; otherwise, the nonparametric Mann–Whitney U test was used. Categorical variables were analyzed using Pearson’s chi-square test or Fisher’s exact test, depending on the expected distribution. Survival outcomes (overall survival, cancer-specific survival, and disease-free survival) were estimated using the Kaplan–Meier method. DR proportions reported in the Results are crude (number of patients with ≥ 1 DR over the study population). Time-to-DR was estimated with the Kaplan–Meier method.

A statistical significance level of 5% (p < 0.05) was established, and 95% confidence intervals were reported when appropriate. Variables with statistical significance or a trend toward significance (p < 0.2) were included in the multivariate analysis, which was performed using logistic regression to estimate the corresponding coefficients and odds ratios (ORs) for selected predictors.

## Results

Between June 2004 and December 2021, a total of 824 patients underwent TEM/TEO at our institution. Based on preoperative indications, patients were distributed into five groups: Group I (n = 536; 65%), Group II (n = 116; 14%), Group III (n = 49; 6%), Group IV (n = 45; 5.5%), and Group V (n = 78; 9.5%).

Of the entire cohort, 104 patients (12.6%) were diagnosed with pT1 rectal ADK on final pathological report, forming the study population.

### Baseline characteristics and preoperative diagnosis (Table 1)

**Table 1:** Demographic and preoperative variables

Demographic and preoperative variables (n = 104)			
Sex (n,%)	Male/Female	60 (57.7)/44 (42.3)	
Age (years) (median, IQR)		71 (15)	
ASA (n,%)	I	4 (3.8)	
	II	44 (42.3)	
	III	48 (46.2)	
	IV	8 (7.7)	
Preoperative biopsy (n,%)	Low-grade ADN	14 (13.5)	
	High-grade ADN	45 (43.5)	
	ADK	45 (43.5)	
Macroscopic tumor size (cm) (median, IQR)		3.5 (1.5)	
Macroscopic tumor size (cm), (ADN biopsy/ADK biopsy)		ADN (n = 59) (median, IQR)	4 (2)
		ADK (n = 45) (median, IQR)	3 (1.5)
Tumor distance to anal margin (cm) (median, IQR)		7 (5)	
ERUS-T staging (n,%)	uT0	10 (9.6)	
	uT1	67 (64.4)	
	uT2	20 (19.2)	
	uT3	2 (1.9)	
	Not assessable	5 (4.8)	
ERUS-T staging (ADN biopsy/ADK biopsy)	ADN (n = 59), (n,%)	uT0	6 (10.2)
		uT1	34 (57.6)
		uT2	16 (27.1)
		uT3	2 (3.4)
		Not assessable	1 (1.7)
	ADK (n = 45), (n,%)	uT0	4 (8,9)
		uT1	33 (73.3)
		uT2	4 (8.9)
		uT3	0 (0)
		Not assessable	4 (8.9)
ERUS-N staging (n,%)	uN0	96 (92.3)	
	uN1	3 (2.9)	
	Not assessable	5 (4.8)	
ERUS-N staging (ADN biopsy/ADK biopsy)	ADN (n = 59), (n,%)	uN0	55 (93.2)
		uN1	3 (5.1)
		Not assessable	1 (1.7)
	ADK(n = 45), (n,%)	uN0	41 (91.1)
		uN1	0 (0)
		Not assessable	4 (8.9)
MRI-T staging (n,%)	mr ≤ 2	70 (67.3)	
	mr3ab	2 (1.9)	
	mr3cd	2 (1.9)	
	Not assessable	2 (1.9)	
	Not performed	28 (26.9)	
MRI-T staging (ADN biopsy/ADK biopsy)	ADN (n = 59), (n,%)	mr ≤ 2	38 (64.4)
		mr3ab	2 (3.4)
		mr3cd	2 (3.4)
		Not assessable	2 (3.4)
		Not performed	15 (25.4)
	ADK(n = 45), (n,%)	mr ≤ 2	32 (71.1)
		mr3ab	0 (0)
		mr3cd	0 (0)
		Not assessable	0 (0)
		Not performed	13 (28.9)
MRI-N staging (n,%)	mrN0	73 (70.2)	
	mrN1	3 (2.9)	
	Not assessable	28 (26.9)	
MRI-N staging (ADN biopsy/ADK biopsy)	ADN (n = 59), (n,%)	mrN0	41 (69.5)
		mrN1	3(5.1)
		Not performed	15 (25.4)
	ADK(n = 45), (n,%)	mrN0	32 (71.1)
		mrN1	0 (0)
		Not performed	13 (28.9)
Lesion type (n,%)	Flat	21 (20.2)	
	Polypoid	24 (23.1)	
	Sessile	51 (49.0)	
	Ulcerated	8 (7.9	
Lesion type (ADN biopsy/ADK biopsy)	ADN (n = 59), (n,%)	Flat	8 (13.6)
		Polypoid	14 (23.7)
		Sessile	35 (59.3)
		Ulcerated	2 (3.4)
	ADK(n = 45), (n,%)	Flat	13 (28.9)
		Polypoid	10 (22.2)
		Sessile	16 (35.6)
		Ulcerated	6 (13.3)
Quadrant location (n,%)	Anterior	28 (26.9)	
	Posterior	25 (24)	
	Right lateral	28 (26.9)	
	Left lateral	23 (22.1)	

ASA = American Society of Anesthesiologists. ADK = adenocarcinoma. ADN = adenoma. ERUS = endorectal ultrasound. MR = magnetic resonance imaging. uT = ultrasound-based T staging. uN = ultrasound-based N staging. mrT = MRI-based T staging. mrN = MRI-based N staging. IQR = interquartile range

Of the 104 patients included, 60 (57.7%) were male, with a median age of 71 years (interquartile range [IQR]: 15 years). Most patients were classified as ASA II (42.3%) or ASA III (46.2%).

In the preoperative endoscopic biopsy, only 45 patients (43.5%) were diagnosed with invasive ADK, highlighting the difficulty of obtaining representative tissue that demonstrates submucosal invasion in superficial lesion sampling.

Preoperative ERUS staged uT0–1 in 77 out of 104 patients, corresponding to an overall diagnostic accuracy of 74%. When stratified by the preoperative biopsy, accuracy for uT0–1 staging was 67.8% (40/59) when the biopsy showed adenoma and 82.2% (37/45) when it showed adenocarcinoma.

Among the 76 patients who underwent MRI, 70 (92.1%) were staged as ≤ T2.

Regarding lesion morphology, the most common pattern was sessile (49%), followed by polypoid (23.1%), flat (20.2%), and ulcerated (7.7%). Tumor distribution across rectal quadrants was relatively homogeneous: anterior and right lateral (26.9% each), posterior (24%), and left lateral (22.1%).

### Surgical, postoperative, and pathological data (Table 2)

**Table 2:** Surgical and postoperative variables (30 days)

Surgical and postoperative variables (30 days) (n = 104)			
Surgical technique (TEM/TEO) (n,%)		52 (50) / 52 (50)	
Surgical time (minutes) (median, IQR)		70 (50)	
Specimen removal (n,%)	En bloc resection	96 (92.3)	
	Fragmented	8 (7.7)	
Defect closure (n,%)	Complete	95 (91.3)	
	Partial	9 (8.7)	
	Open	0 (0)	
Hospital stay (days) (median, IQR)		3 (2)	
Overall morbidity (n,%)		24 (23.1)	
Clavien-Dindo Morbidity (n,%)	0	80 (76.9)	
	I	16 (15.4)	
	II	3 (2.9)	
	IIIa	1 (1)	
	IIIb	3 (2.9)	
	IVa	0 (0)	
	IVb	1 (1)	
	V	0 (0)	
Significant Clavien-Dindo Morbidity (> II) (n,%)		5 (4.8)	
Anatomopathological Results	Tumor size (cm) (median, IQR)	3.55 (1.5)	
	Tumor size ADK (cm) (median, IQR)	1.2 (1.6)	
	ADN over ADK (n,%)	76 (73.1)	
	Type of ADN over ADK (n = 76) (n,%)	Serrated	1 (1.3)
		Tubular	3 (3.9)
		Tubulovillous	18 (23.7)
		Villous	54 (71.1)
	Resection margin > 1 mm from ADK (n,%)	Free	96 (92.3)
		In contact with ADK	2 (1.9)
		Not assessable (fragmented)	6 (5.8)
	Lateral Margin (mm) (median, IQR)	6 (5)	
	Deep Margin (mm) (median, IQR)	6 (4)	
	ADK differentiation grade (n,%)	Well differentiated	96 (93.3)
		Moderately differentiated	4 (3.8)
		Poorly differentiated	3 (2.9)
		Undifferentiated	1 (1)
	Presence of Lymphatic Invasion (n,%)	7 (6.7)	
	Presence of Vascular Invasion (n,%)	2 (1.9)	
	Presence of Perineural Invasion (n,%)	4 (3.8)	
	Presence of Lymphocytic Infiltrate (n,%)	7 (6.7)	
	Presence of Budding (n,%)	1 (1)	
	Presence of any poor prognostic factor (n,%)	13 (12.5)	

TEM = transanal endoscopic microsurgery. TEO = transanal endoscopic operation. IQR = interquartile range. ADK = adenocarcinoma. ADN = adenoma

The surgical techniques TEM and TEO were used in equal proportion, with 52 patients each (50%). The median operative time was 70 min (IQR: 50). En bloc resection was achieved in 96 of the 104 cases (92.3%), and complete closure of the rectal wall defect was performed in the vast majority of patients (91.3%).

The median length of hospital stay was 3 days (IQR: 2). Overall morbidity was 23.1%, with minor complications being the most frequent (Clavien–Dindo grade I: 15.4%; grade II: 2.9%). Only five patients (4.8%) experienced complications classified as Clavien–Dindo ≥ III, considered clinically significant.

Thirteen patients (12.5%) presented at least one adverse histopathological feature, including lymphatic, vascular, or perineural invasion, lymphocytic infiltration, or tumor budding (Supplementary Information-Table 1). Among them, four patients underwent completion TME: three via low anterior resection (LAR) and one via abdominoperineal resection (APR). An additional three patients underwent completion TME for other reasons, in two of these, ADK was present at the resection margins (one LAR and one APR). In the third case, although the preoperative biopsy indicated an adenoma, both ERUS and MRI suggested metastatic lymph nodes. Final pathological analysis of the specimen after TEM/TEO revealed invasive ADK, and the patient subsequently complete APR. The remaining nine patients with adverse pathological features declined completion TME. Therefore, a total of seven patients (6.7%) in the series underwent completion surgery with TME. In summary, of the 104 patients initially included, 13 were excluded due to high-risk pathological features requiring completion TME, and 3 additional patients underwent completion TME for other indications, resulting in a final cohort of 88 patients included in the oncological outcome analysis.

### Follow-up and oncological outcomes

#### Primary outcome: local recurrence (LR) (Fig. 1)

**Fig. 1:**
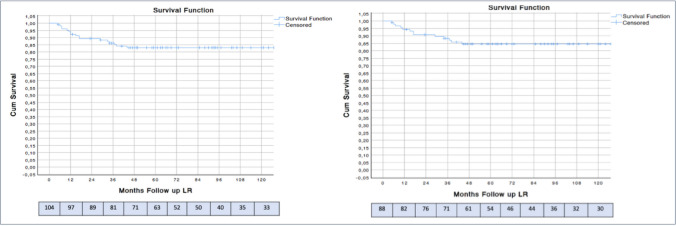
Kaplan–Meier curves for local recurrence-free survival after local excision (LE) for pT1 rectal adenocarcinoma. Curves are shown for the entire cohort of 104 patients (left) and the subcohort of 88 patients (right) without adverse pathological features or positive margins. The final cohort comprised 88 patients (104 initially treated – 13 high-risk tumors – 3 completion TME for other indications)

The median follow-up was 91 months (IQR: 84). The overall LR rate in the study cohort was 16.3% (17 out of 104 patients), with time to onset ranging from 8 to 44 months after surgery (Fig. 1).

Among the four patients with adverse histopathological features who underwent completion TME, one developed LR. This patient was successfully salvaged with curative intent by APR. The remaining three patients did not experience LR during follow-up.

Among the nine patients with adverse histological features who declined completion TME, three developed LR. Two of them underwent curative surgery via APR, while one received palliative treatment.

In the cohort of 88 patients without adverse histopathological features who did not undergo completion TME, 13 patients developed LR (14.8%). Of these, 11 were treated with curative intent through radical surgery (TME), whereas two were not candidates for curative treatment (Supplementary Information-Table 2).

#### Distant recurrence and survival outcomes

**Fig. 2:**
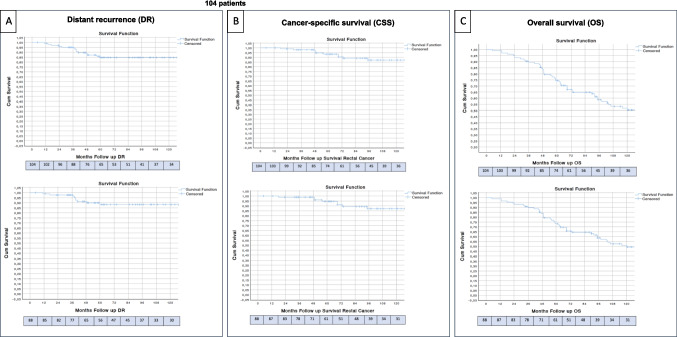
Kaplan–Meier curves for long-term oncological outcomes: (A) distant recurrence (DR), (B) rectal cancer–specific survival (CSS), and (C) overall survival (OS) in the full cohort of 104 patients (top) and the subcohort of 88 patients (bottom) without adverse pathological features or positive margins. Panel A shows the Kaplan–Meier estimate of time to distant recurrence (DR). DR percentages reported in the Results are crude proportions

Crude DR was observed in 14 of the 104 patients (13.5%), with a time of onset ranging from 11 to 56 months after surgery (Fig. 2A). In eight of these cases, DR occurred concurrently with LR (7.7%). Overall, 24 patients (23.1%) experienced some form of recurrence—either local, distant, or both.

In the subgroup of 88 patients without adverse histopathological features who did not undergo completion TME, nine patients (10.2%) developed DR, of whom six (6.8%) presented it in combination with LR. Detailed information on cases with LR and DR is provided in Supplementary Information–Table 2.

Rectal cancer–specific mortality following TEM/TEO was 9.3% (9 out of 97 patients). Rectal cancer–specific survival was 94%, and overall survival reached 74%.”. These outcomes are illustrated in Fig. 2.

#### Analysis of factors associated with local recurrence

A univariate analysis was performed to identify variables associated with LR in the cohort of 88 patients treated exclusively with TEM/TEO, without adverse histopathological features or involved margins. The results of this analysis in relation to LR are presented in Table 3.

**Table 3:** Univariate analysis of local recurrence in patients without risk factors for Local Recurrence (LR), (n = 88)

Variables			No LR (n = 75)	LR (n = 13)	p-value (95% CI)
Sex (n,%)	Female		30 (85.7)	5 (14.3)	0.99 1.71
	Male		45 (84.9)	8 (15.1)	
Age (years) (median, IQR)			71 (15)	72 (14)	0.8
ASA (n,%)	I-II		34 (87.2)	5 (12.9)	0.77
	III-IV		41 (83.7)	8 (16.3)	
Preoperative biopsy (n,%)	ADN Low grade		9 (75)	3 (25)	0.48
	ADN High grade		33 (84.6)	6 (15.4)	
	ADK		33 (89.2)	4 (10.8)	
Macroscopic tumour size (cm) (median, IQR)			3.5 (1)	5 (3)	0.09
Distance of tumour to anal margin (cm) (median, IQR)			7 (4)	6 (5)	0.3
Distance of tumour to anal margin (low rectum/medium–high rectum) (n,%)	Low rectum (≤ 5 cm)		26 (81.3)	6 (18.8)	0.54
	Mid-high rectum (> 5 cm)		49 (87.5)	7 (12.5)	
Type of lesion (n,%)	Flat		14 (77.8)	4 (22.2)	0.23
	Polypoid		18 (94.7)	1 (5.3)	
	Sessile		40 (87)	6 (13)	
	Ulcerated		3 (60)	2 (40)	
Polypoid-sessile vs. flat-ulcerated lesion (n,%)	Polypoid-sessile		58 (89.2)	7 (10.8)	0.09
	Flat-ulcerated		17 (73.9)	6 (26.1)	
Location by quadrants (n,%)	Anterior		20 (80)	5 (20)	0.41
	Posterior		20 (95.2)	1 (4.8)	
	Right lateral		20 (80)	5 (20)	
	Left lateral		15 (88.2)	2 (11.8)	
Surgical technique (n,%)	TEM		39 (90.7)	4 (9.3)	0.23
	TEO		36 (80)	9 (20)	
Surgical time (min) (median, IQR)			65 (46)	100 (75)	0.08
Excision of the specimen (n,%)	En bloc resection		72 (87.8)	10 (12.2)	0.04
	Fragmented		3 (50)	3 (50)	
Suture of the defect (n,%)	Complete		71 (88.8)	9 (11.3)	0.02
	Partial		4 (50)	4 (50)	
	Open		0	0	
Global Mobility (n,%)	No		54 (81.8)	12 (18.2)	0.17
	Yes		21 (95.5)	1 (4.5)	
Significant Clavien-Dindo morbidity (> II) (n,%)	Clavien-Dindo relevant ≤ II		70 (84.3)	13 (15.7)	0.59
	Clavien-Dindo relevant > II		5 (100)	0 (0)	
ADK tumour size (cm) (median, IQR)			1.25 (1.6)	1.1 (1.6)	0.73
ADN over ADK	ADN over ADK		55 (85.9)	9(14.1)	0.74
	ADK		20 (83.3)	4 (16.7)	
Type of ADN over ADK (n = 70)	Serrated		0 (0)	1 (100)	0.16
	Tubular		3 (100)	0 (0)	
	Tubulovillous		10 (83.3)	2 (16.7)	
	Villous		42 (87.5)	6 (12.5)	

LR = local recurrence. ASA = American Society of Anesthesiologists classification. ADN = adenoma. ADK = adenocarcinoma. IQR = interquartile range. TEM = transanal endoscopic microsurgery. TEO = transanal endoscopic operation. Min = minutes. Clavien–Dindo > II = clinically significant postoperative complications (grade III or higher). ADN over ADK = presence of adenomatous component overlying adenocarcinoma in the same lesion

Tumor size, lesion type (flat-ulcerated vs. polypoid-sessile), operative time, complete vs. partial defect closure, and en bloc resection showed statistical significance or a trend toward significance. These variables were entered into a binary logistic regression model using the forced-entry (enter) method.

The multivariate analysis revealed that only the flat-ulcerated lesion type was significantly associated with LR (p = 0.01), with an odds ratio of 6.8 and a 95% confidence interval of 1.5 to 30.4.

## Discussion

In this study involving 104 patients with pT1 rectal adenocarcinoma treated by local LE using TEM/TEO, the overall LR rate was 16.5%, and 14.8% among the 88 patients without adverse histopathological features. These figures, although within the range reported in the literature, confirm that LR remains a relevant clinical challenge even in apparently low-risk cases.

Local recurrence after LE of pT1 rectal cancer shows wide variability in the literature, raising concerns about the oncological safety of this treatment strategy. Traditionally, a recurrence rate below 10% has been considered a clinically acceptable threshold [27, 28], although TME achieves rates below 5% [29]. This greater tolerance for LR is justified by the advantages of LE, including lower surgical complexity, reduced postoperative morbidity, less impact on sphincter and genitourinary function, and improved quality of life compared to TME [30, 31]. However, variability in recurrence rates becomes more pronounced when analyzing larger pT1 rectal tumors (> 2 cm), which are typically candidates for resection via TEM/TEO. Major clinical guidelines, such as those from the Japanese Society for Cancer of the Colon and Rectum (JSCCR) [32] and the European Society of Gastrointestinal Endoscopy (ESGE) [33], define high-risk tumors as those with submucosal invasion ≥ 1000 µm, lymphovascular invasion, poor differentiation, and the presence of tumor budding. To these criteria, the achievement of an en bloc resection with tumor-free margins—ideally greater than 1 mm—is often added, although this latter threshold remains under debate [10].

Our study represents one of the largest and most homogeneous series published to date, comprising 104 patients operated on at a single center by the same surgical team, using standardized criteria for therapeutic indication, surgical technique (full-thickness excision without penetration into the perirectal fat [16, 22]), decision-making regarding completion TME, and a unified follow-up protocol. These findings illustrate the inherent limitations of preoperative staging in early rectal lesions, yet our ERUS accuracy (74%) falls within the range reported for pT1 tumors. Similar figures were observed in our previous prospective study analyzing ERUS performance in candidates for TEM, which reported an overall accuracy of 78% [17]. In this cohort, MRI primarily served to rule out locally advanced (≥ T3) tumors and to assess nodal status, consistent with its known limitations in distinguishing T1 from T2 lesions. Therefore, its high proportion of ≤ T2 staging should be interpreted as confirmation of its value for excluding advanced disease rather than for differentiating early stages.

Deep submucosal invasion was not systematically evaluated as a risk factor during the study period, as there was no standardized measurement method in pathology reports at that time. In 2021, our group proposed a new approach based on the quantitative assessment of residual healthy submucosa [34], which has since been adopted in our institutional practice and was recently validated with excellent interobserver agreement [35].

In the present study involving 104 patients, LR rate was 16.5%. Among patients without adverse histopathological features, LR occurred in 13 out of 88 patients (14.8%). This relatively high rate had already been observed in our previous study [34], where, with a shorter follow-up period, an LR rate of 13.8% was reported in a cohort of 80 patients.

In an attempt to identify potential explanatory variables beyond classical pathological features and resection margins [32, 33], a univariate analysis followed by logistic regression was performed. This analysis identified flat-ulcerated morphology as a possible factor associated with LR. This variable has not been previously recognized as significant in earlier studies [28].

Given the absence of clearly established clinical predictors and the low prevalence of high-risk pathological features in pT1 rectal cancer, accurate assessment of the depth of submucosal invasion becomes particularly relevant. Although the prognostic value of this variable remains a topic of considerable debate [7, 36], there is evidence that in cases of superficial submucosal invasion—such as sm1 according to the Kikuchi classification (up to 200–300 µm) [37], the risk of lymph node involvement is around 3–4%. However, when the ADK extends into the muscularis propria (pT2), the risk of nodal involvement—and consequently, LR—exceeds 20% [11]. These findings support the hypothesis that the depth of submucosal invasion plays a key role in locoregional spread and, therefore, in the risk of LR.

When applying standard classification systems for ADK submucosal invasion—both qualitative and quantitative—certain biases may be introduced that are rarely addressed. One of the most relevant is the high variability in the total thickness of the submucosa, meaning that absolute invasion values may correspond to either superficial or deep regions, each with distinct oncological implications. Another critical issue is the method used to measure from the surface, which is hindered by the absence of a fixed anatomical landmark such as the muscularis mucosae, present in fewer than 30% of cases. This limitation compromises the accuracy of measurement and, consequently, the reliability of invasion analysis [16, 34].

Given these limitations, we proposed an alternative, more reliable and reproducible approach [34]: measuring from an anatomically stable structure, such as the muscularis propria, in the resected specimen of pT1 rectal cancer—evaluating the residual healthy submucosa as a prognostic selection marker. This method requires full-thickness excision of the rectal wall, without the need to penetrate the mesorectal fat [34]. The TEM/TEO technique, by enabling precise transmural excision below the muscular layer, ensures an adequate specimen for this analysis. Furthermore, in cases requiring additional surgery by TME, it preserves the integrity of the mesorectum [25]. Recent studies on transmuscular endoscopic resections—through the muscularis propria— are also appropriate for this purpose [38].

It should be noted that, in addition to LR, DR occurred in 13.5% of patients, and 23.1% developed some form of recurrence—either LR, DR, or both. Therefore, a promising strategy to reduce locoregional spread could be the administration of neoadjuvant therapy. In a recent study, patients with rectal ADK T2–T3ab, N0, M0 treated with neoadjuvant therapy followed by TEM showed an LR rate of 7.4% and a DR rate of 12.3%, compared to TME [39]. These figures, lower than those observed in our study and others previously published, may suggest a potential benefit of this strategy even in pT1 rectal cancer.

The cancer-specific survival rate of 94% and the overall survival rate of 74% observed in our study are consistent with findings from other series of patients treated with TEM/TEO and long-term follow-up [12, 28]. Leijtens et al. reported a 5-year cancer-specific survival rate of 91.7% [13]. Similarly, two recent meta-analyses reported comparable 5-year cancer-specific survival rates of 91.4% and 93%, respectively, in patients treated with LE [12, 28]. While these outcomes are favorable, they highlight the importance of optimizing patient selection in order to reduce the risk of LR and avoid compromising long-term survival.

Among the main limitations of this study are its retrospective, single-center design and the absence of a comparator group treated with TME. Nevertheless, the sample size, prolonged follow-up period, and the high degree of homogeneity in both the surgical technique and the surgical team responsible for all procedures strengthen the robustness and consistency of the findings.

## Conclusions

Local recurrence of pT1 rectal adenocarcinoma remains a clinical challenge, even in the absence of adverse histopathological features. In our series, under these conditions, the recurrence rate reached 14.8%, highlighting the need to redefine current selection criteria and incorporate new diagnostic and therapeutic tools. Future multicenter studies with standardized criteria and a focus on quantifying submucosal invasion will be essential to advance toward a more precise and personalized treatment approach for these patients.

## Supplementary Information

Below is the link to the electronic supplementary material.

Supplementary file1 (XLSX 16.3 KB)

Supplementary file2 (XLSX 13.5 KB)

## Data Availability

The full dataset supporting the findings of this study is provided as a supplementary Excel file submitted alongside the manuscript (Supplementary Information\_Table 3. pT1 TAULI-T1 casuistic\_def-rev). Further information is available from the corresponding author upon reasonable request.

## Declarations

### Permission to reproduce material from Other sources

No permission was required to reproduce material from other sources.
